# Time Series Data Mining for Linking the Shape of Bacterial Growth Curves to Biological Functions

**DOI:** 10.34133/csbj.0029

**Published:** 2026-04-01

**Authors:** Zehui Lao, Bei-Wen Ying

**Affiliations:** ^1^ School of Life and Environmental Sciences, University of Tsukuba, 1-1-1 Tennodai, Tsukuba 305-8572, Ibaraki, Japan.; ^2^ MiCS, University of Tsukuba, 1-1-1 Tennodai, Tsukuba 305-8572, Ibaraki, Japan.

## Abstract

Connecting bacterial growth dynamics to biological functions is essential for understanding microbial systems, yet studies directly examining growth dynamics remain limited. Here, we analyzed over 10,000 growth curves from single-gene knockout *Escherichia coli* strains using combined dynamic time warping and derivative dynamic time warping methods, followed by hierarchical clustering based on shape similarity with and without considering experimental replicates. Clustering revealed groups enriched for specific gene categories and biological processes, particularly enzymes and biosynthesis pathways. Growth curves with high reproducibility were associated with conserved biosynthetic functions. These findings demonstrate that time series data mining can effectively link bacterial growth dynamics to biological functions, providing a framework for interpreting complex genetic effects on population behavior and advancing data-driven approaches in biotechnological and microbial research.

## Graphical Abstract

**Figure:**
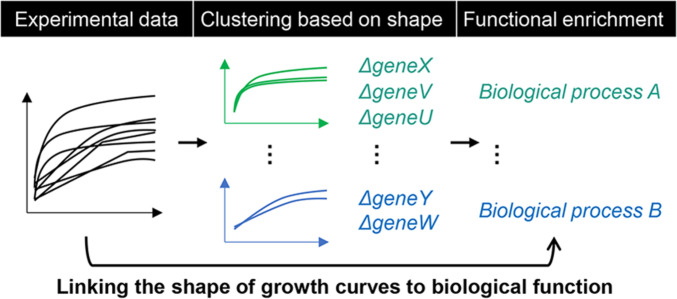

## Introduction

Bacterial growth dynamics describe the changes in bacterial population size over time, typically encompassing the lag, exponential, stationary, and death phases [1,2]. These dynamics provide detailed insights into how bacteria respond to environmental and genetic changes [3,4]. Growth curves, which represent time series measurements of optical density (OD, a proxy for cell density) [5–7], are commonly used to depict these dynamics. While typical growth curves are often analyzed using S-shaped models such as the Logistic and Gompertz functions [8,9], actual bacterial population dynamics are frequently more complex and cannot always be fully captured by these simplified models [10,11]. Growth dynamics reflect the integrated output of various cellular processes [12,13], offering insights into how genetic and environmental factors influence survival and adaptation [14,15]. For example, changes in lag time or growth rate can indicate metabolic bottlenecks or stress responses [16,17], and a secondary exponential phase may result from a switch in carbon sources [18,19]. Therefore, understanding the diverse patterns of bacterial growth curves is essential for accurately interpreting bacterial behavior and managing microbial communities [20,21].

A fundamental goal in understanding bacterial growth is to link biological functions to overall population dynamics as represented by growth curves. However, establishing such connections has been challenging due to limitations in computational methods and the availability of comprehensive experimental datasets. Traditional approaches, such as curve-fitting and computational simulations, have primarily been used to validate hypotheses or interpret results [22,23], rather than to uncover novel biological relationships. Previous studies have often focused on the effects of genetic or environmental factors on specific growth parameters [24,25], rather than on the entire growth dynamics. Recently, the application of machine learning methodologies to biological studies [26,27], combined with the increasing availability of high-throughput bacterial growth data [4,15,28], has enabled more data-driven approaches to explore growth dynamics [29]. These advances in both analytical methods and data resources now make it possible to connect growth curves to biological functions, offering more profound insights into dynamic living systems.

In this study, we aimed to identify potential links between biological functions and bacterial growth dynamics. To introduce genetic variation for association analyses, we utilized growth curves from single-gene knockout strains [30] obtained in our previous work [28]. These growth curves were analyzed using data mining techniques to capture overall growth patterns, as reducing the dimensionality of the data may obscure important features in large-scale model training [31,32]. Because growth curves are temporal records of bacterial population size, we assessed the similarity between pairs of curves using machine learning algorithms designed for time series analysis [33,34]. Specifically, dynamic time warping (DTW) and derivative dynamic time warping (DDTW) served as the distance algorithms to accurately measure similarities based on the shape of growth curves. Based on these distances, hierarchical clustering was then used as the unsupervised learning method to classify the curves into meaningful biological groups. The curves were then clustered based on their shape similarities, using strategies that either relied solely on statistical criteria or incorporated information from experimental replicates. Since biological data often include experimental replicates with unavoidable errors, especially when working with living cells [35,36], we also examined clustering approaches that account for replicate variance to better understand how data mining can leverage biological experimental data. In summary, this study explores the feasibility of linking bacterial growth curves to biological functions and demonstrates how data mining approaches can be applied to large-scale biological datasets.

## Results

### Workflow of the data mining for linking bacterial growth curves to biological functions

**Fig. 1.**
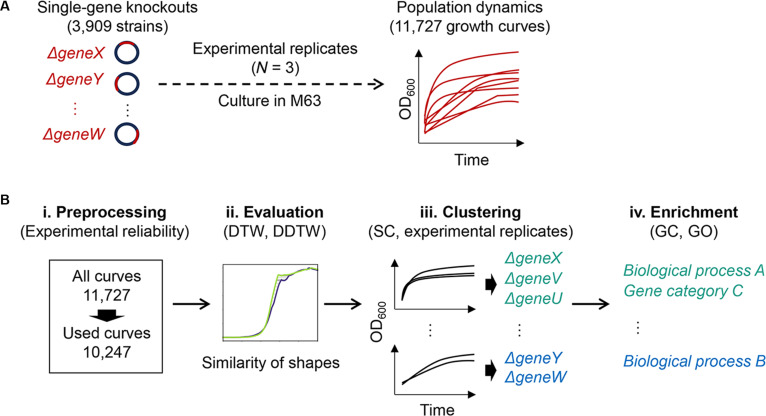
Overview of bacterial growth curve data mining. (A) Data collection. The process for gathering bacterial growth curve data is shown. It indicates the differences among *E. coli* strains (genotypes), growth conditions, experimental replicates, and the total number of growth curves. (B) Data analysis. A step-by-step summary of the data mining process for the growth curves from (A) is provided. The methods for time series analysis, clustering, and functional enrichment are included.

The dataset of bacterial growth curves was deposited in the database *figshare* (28342043) [37]. A total of 11,727 growth curves from 3,909 single-gene knockout *Escherichia coli* strains (each with 3 experimental replicates) cultivated in the minimal medium M63 were used in the present study (Fig. 1A). Since these genes were successfully deleted from the wild-type (WT) genome, they were considered nonessential genes [30,38]. The growth curves depicted the temporal changes in bacterial population size, with OD_600_ serving as a proxy for cell density. The OD_600_ measurements were recorded over 24 to 48 h at 15-min intervals, resulting in 48 to 96 data points per growth curve.

The data mining process was conducted as shown in Fig. 1B. Growth curves with no growth (zero) or incomplete growth phases were discarded, resulting in 10,247 curves for subsequent analysis (Fig. 1B, i). Note that this preprocessing step was conducted to ensure the biological validity of the experimental dataset. Pairwise similarities between the growth curves were calculated using 2 methods: DTW [39,40] and DDTW [41,42]. To account for the distinct features of each method, DTW and DDTW were combined. This enabled each growth curve to be assigned a score quantifying its resemblance to every other curve (Fig. 1B, ii). The resulting DTW and DDTW metrics were then used to cluster the growth curves, with the optimal number of clusters determined based on statistical or experimental criteria (Fig. 1B, iii). Ultimately, functional enrichment of the knockout genes assigned in each cluster was performed to identify any gene categories or biological processes relating to specific growth patterns (Fig. 1B, iv).

#### Clustering growth curves using only statistical criteria

**Fig. 2.**
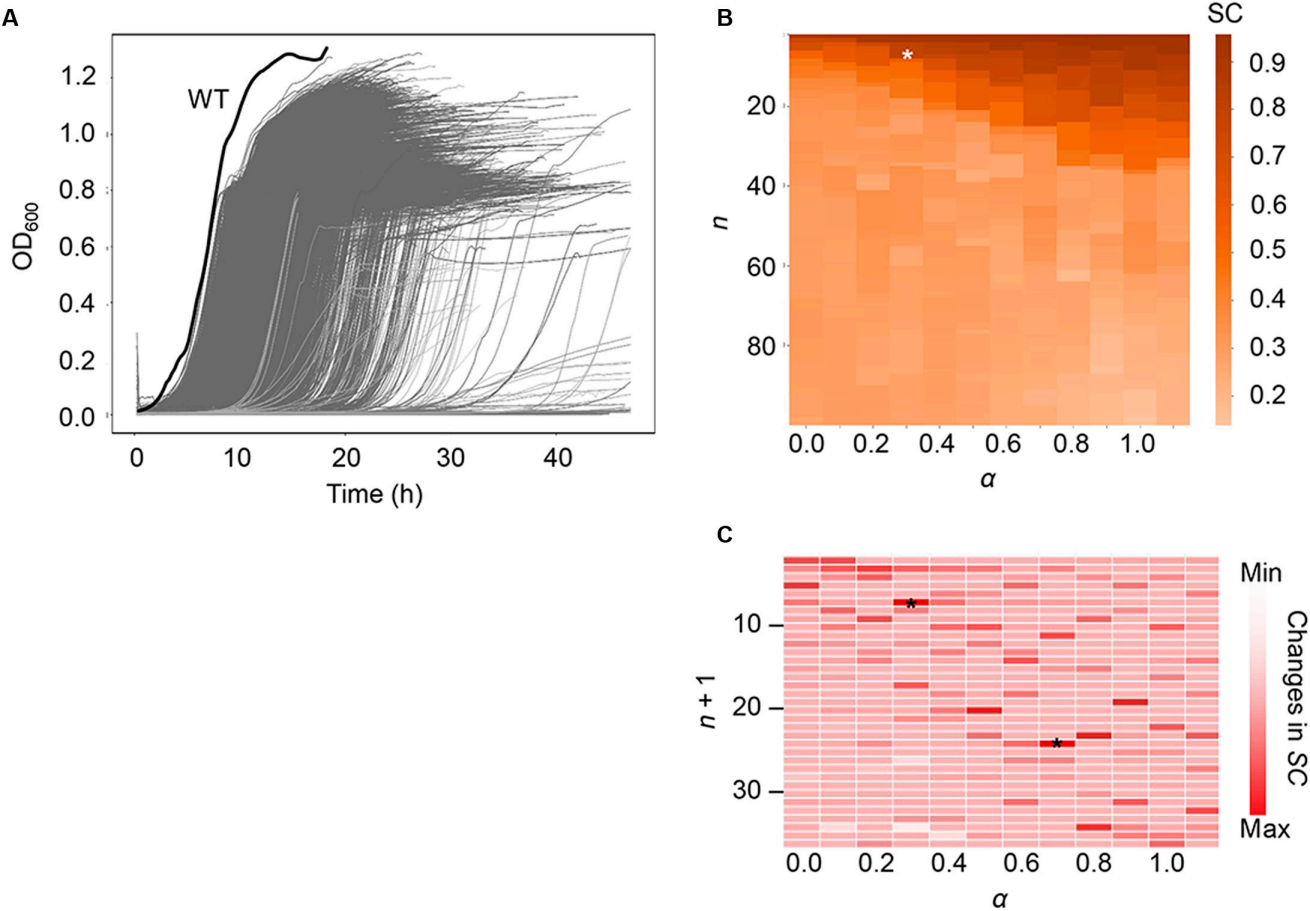
Statistical evaluation of the similarity of growth curves. (A) Preprocessed growth curves. A total of 3,880 growth curves corresponding to 3,880 single-gene knockouts are shown in gray. The wild-type strain is indicated in a bold black line. (B) Similarity of growth curves based on the silhouette coefficient (SC). Orange shading indicates the SC values. *α* and *n* represent the weight bias between dynamic time warping (DTW) and derivative dynamic time warping (DDTW), and the number of clusters, respectively. An asterisk marks the highest SC value in the heatmap. (C) Heatmap showing changes in SC. Red shading represents the change in SC as the number of clusters increases, indicated by *n* + 1. Asterisks denote the optimal combination of *α* and *n*.

For the time series analysis, one growth curve from each set of 3 experimental replicates was selected, resulting in 3,880 growth curves corresponding to 3,880 deleted genes (Fig. 2A and Table S1). All curves underwent several preprocessing steps to ensure accuracy and consistency (see Materials and Methods). As the traditional models failed to categorize nongrowing curves, the analytical framework based on the shape of growth curves was required. As a supplementary comparison, the 3,880 growth curves were fitted with the well-established Gompertz and logistic models, and the resultant specific growth rate and carrying capacity were subjected to K-means clustering. A severe misclassification was observed (Fig. S1), that is, a massive number of nongrowing curves in the regular growth cluster. Rigid S-curve equations are unsuitable for fitting flat or highly aberrant trajectories, which are common in bacterial growth curves. To overcome these artifacts and accurately capture complex population dynamics, DTW and DDTW were used to assess the shape similarity of the growth curves, converting the time series data into single similarity values (Tables S2 and S3). A hyperparameter, *α*, was introduced to balance the contributions of DTW and DDTW, as these methods emphasize different aspects of similarity [43]. The number of clusters (*n*) and *α* were varied across broad ranges (*n*: 2 to 100; *α*: 0 to 1). The optimal combination was determined using the silhouette coefficient (SC), with higher values indicating better clustering. Increasing the number of clusters led to a decrease in SC (Fig. 2B and Table S4), suggesting that fewer clusters were preferable. The most notable decreases in SC occurred at *α* = 0.3, *n* = 7, and *α* = 0.8, *n* = 24 (Fig. 2C), as identified by the elbow method [44]. Thus, the optimal number of clusters for categorizing the 3,880 growth curves was determined to be either 7 or 24.

#### Enrichment of particular biological functions in the 2 statistically identified clusters

**Fig. 3.**
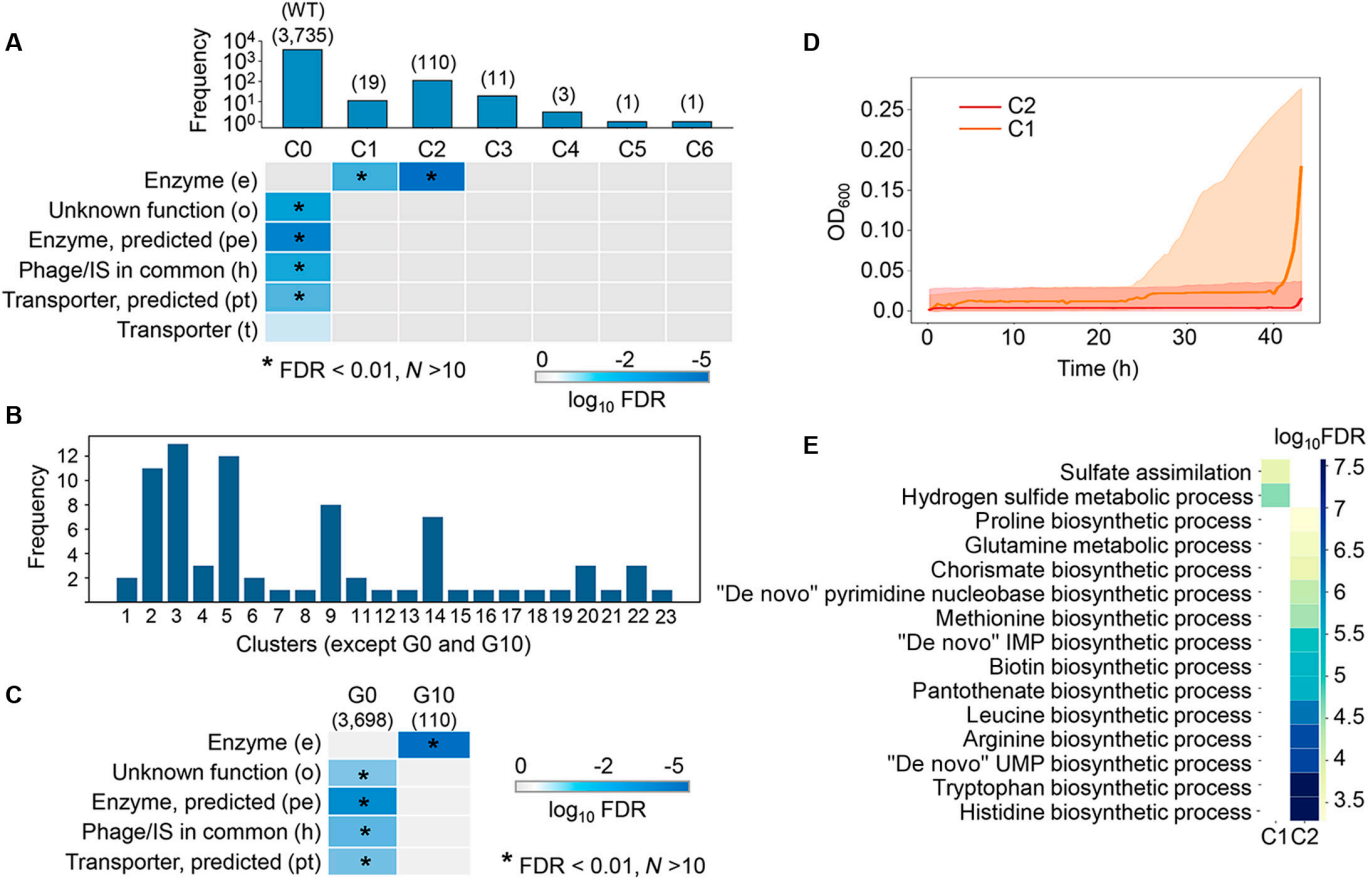
Enriched biological functions of the clustered growth curves. (A) Enriched gene categories in the 7 clusters defined by statistical criteria. The number of growth curves (knockout genes) assigned to each cluster is shown as a bar graph, with the exact count indicated. The wild-type (WT) strain is indicated, representing the biological baseline. Statistically significant gene categories are highlighted, with their significance levels indicated by blue shading. Asterisks indicate high significance. (B) Number of growth curves assigned to 24 clusters, with the 2 largest clusters omitted. (C) Enriched gene categories in the 2 largest clusters. The number of growth curves (knockout genes) assigned to each cluster is shown. No significant enrichment was observed in the other 22 clusters. Statistically significant gene categories are highlighted, with their significance levels indicated by blue shading. Asterisks indicate the high significance. (D) Averaged trajectories of C1 and C2. Light and dark orange indicate C1 and C2, respectively. Lines show the mean growth curve within each cluster. Transparent shaded bands illustrate the pointwise min–max envelope of all curves in the respective cluster, with each curve truncated to the first 90% of time points. The lower and upper bounds were calculated as the minimum and maximum at each time point. (E) Enriched GO in the 7 clusters. Statistically significant Gene Ontology (GO) terms are highlighted, with their significance levels shown by yellow–blue shading.

While these growth curves were divided into 7 clusters (C0 to C6), most were assigned to the same cluster, C0 (Fig. 3A). It mainly represented typical population dynamics similar to the WT growth curve (Fig. 2A). By projecting the WT growth curve onto the clustering framework, it was consistently assigned to C0 (Fig. 3A), suggesting this cluster served as the biological baseline for normal growth curves. Increasing the number of clusters from 7 to 24, the alternative optimal combination did not improve the differentiation among growth curves, remaining 2 major clusters (G0 and G10) (Fig. 3B). The largest cluster out of either 7 (C0) (Fig. 3A) or 24 clusters (G0) (Fig. 3C) significantly enriched 4 gene categories of unknown function (o), predicted enzyme (pe), phage/IS in common (h), and predicted transporter (pt). All of these categories have unclear functions, so their impact on the growth curves might be too subtle to distinguish.

Functional enrichment failed to detect any significant gene categories in 4 out of the 7 clusters, while the enzyme was commonly enriched in 2 clusters, C1 and C2 (Fig. 3A and Table S6). The growth curves in C1 exhibited a severely prolonged lag phase but eventually reached a normal carrying capacity, while those in C2 showed almost no growth or lethality (Fig. 3D). The remaining minor clusters (C3 to C6) represented rare, highly aberrant trajectories without statistically significant functional enrichment. This indicated different patterns of enzymes involved in the bacterial population dynamics. Gene Ontology (GO) enrichment further identified biological processes related to amino acid biosynthesis and sulfur metabolism in C1 and C2, respectively (Fig. 3E). It suggested that the enzymes involved in these biological processes affected the bacterial growth curves differently, aligning with previous studies that reported leucine as a growth decision maker [15] and sulfur’s role in the extended lag phase [45,46]. The 15 knockouts clustered in C2 exhibited a partially lethal effect on bacterial growth (Fig. 3D, red), which was reasonable since the growth curves were obtained in minimal medium without any amino acid supplement. The knockouts clustered in C1, such as *cysA*, *cysC*, *cysD*, *cysH,* and *cysW* (Table S6), which are responsible for transporting sulfate [47–49], allowed exponential growth but caused an extensive growth delay (Fig. 3D, orange). Such a long lag phase might be owing to partial compensation by magnesium sulfate and ammonia sulfate supplied in the medium. In brief, as a first pilot trial, the purely statistical approach identified associations between bacterial population dynamics and biological functions at the category or pathway levels.

#### Clustering growth curves while accounting for experimental replicates

**Fig. 4.**
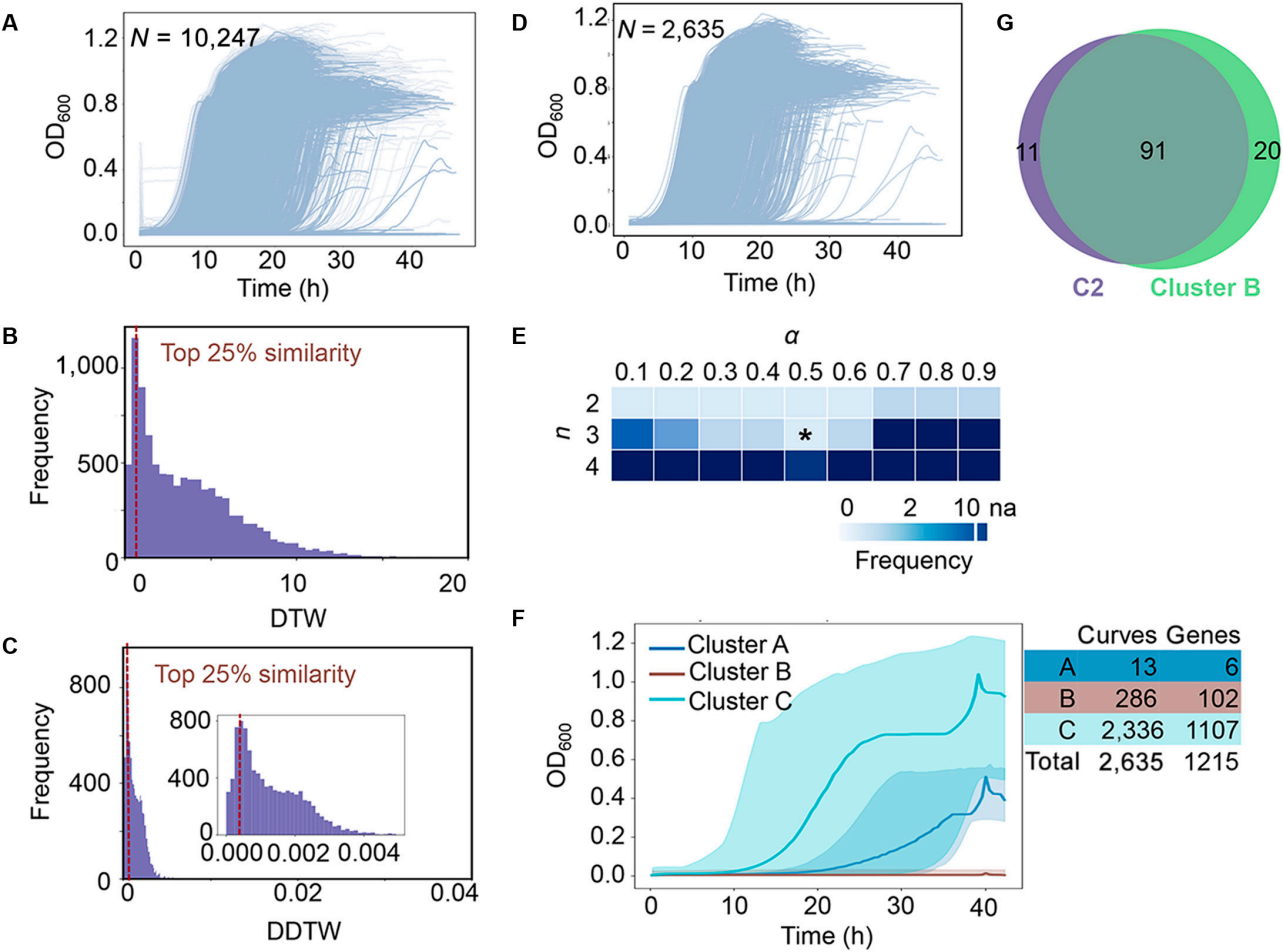
Clustering growth curves considering experimental replicates. (A) 10,247 of preprocessed growth curves. (B) Histogram of within-replicate DTW distances. The dashed vertical line marks the DTW threshold value of 1.105, referring to the top 25%. (C) Histogram of within-replicate DDTW distances. The dashed vertical line indicates the DDTW threshold value of 0.000506, representing the top 25%. The inset provides a close-up view. (D) 2,635 growth curves after data cleaning. (E) The mean frequency of experimental replicates assigned to different clusters. The number of growth curves, with experimental replicates grouped into different clusters, was counted. The moving average of 3 neighboring counts was calculated as the mean frequency. *α* and *n* represent the weight bias between DTW and DDTW, and the number of clusters, respectively. An asterisk indicates the best combination (*α* = 0.5, *n* = 3). (F) Averaged trajectories of the 3 clusters. Color variation indicates the 3 clusters. Lines show the mean growth curve within each cluster. Transparent shaded bands illustrate the pointwise min–max envelope of all curves in the respective cluster, with each curve truncated to the first 90% of time points. The lower and upper bounds were calculated as the minimum and maximum at each time point. The number of growth curves assigned to each cluster is shown in the table. (G) Venn diagram illustrating overlaps between C2 and Cluster B. The number of growth curves is shown.

Substantial variance in experimental replicates is a common feature of biology. Whether such a feature could be adopted as an indicator for clustering the growth curves obtained in the laboratory was examined. A total of 10,247 processed growth curves containing experimental replicates were used (Fig. 4A and Table S7). Data cleaning was explicitly performed to remove growth curves that were substantially different from others obtained under identical experimental conditions (experimental replicates). Pairwise similarities within the same experimental group were computed using DTW (Table S8) and DDTW (Table S9). The growth curves that simultaneously satisfied the top 25% similarities for both DTW (Fig. 4B) and DDTW (Fig. 4C) were selected, which resulted in 2,635 growth curves from 1,215 single-gene knockout strains (Fig. 4D and Table S10).

The DTW and DDTW metrics weighted by the hyperparameter, *α*, were applied as shown above (Fig. 2); however, the optimal number of clusters was determined using a rule that ensured experimental replicates remained in the same cluster. This rule guaranteed that genetically and environmentally identical growth curves were grouped together, as they were expected to have similar shapes. The combination of *n* and *α* indicated that increasing the number of clusters more frequently separated the growth curves of replicates into different clusters, regardless of changes in *α* (Fig. 4E). According to the rule, the best combination of *n* and *α* was at *α* = 0.5 and *n* = 3, representing the largest number of clusters with the lowest tendency to separate. Consequently, 3 clusters were identified, each containing a vastly different number of growth curves across the respective knockout genes (Fig. 4F), in which the experimental replicates were all divided into the same cluster (Table S11). The absence of the 102 genes in Cluster B somewhat had a lethal effect on bacterial growth. These genes largely overlapped with the genes assigned in C2 (Fig. 4G), demonstrating the consistency of the clustering dependent on statistics and considering experimental replicates.

#### Enriched functions in the clusters determined by considering experimental replicates

**Fig. 5.**
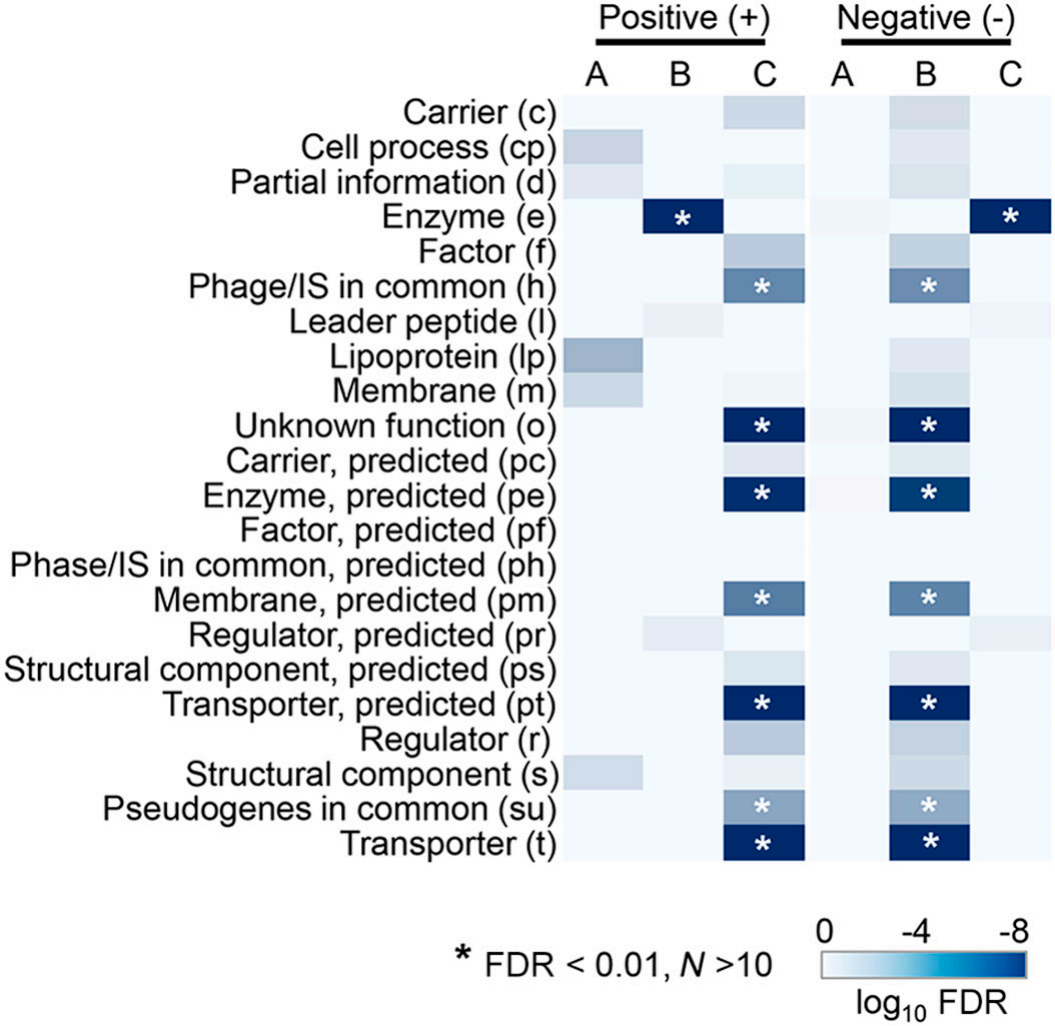
Positively and negatively enriched functions in the 3 clusters. The number of growth curves (knockout genes) assigned to each cluster is shown. Statistically significant gene categories are highlighted, with their significance levels indicated by blue shading. Asterisks indicate high significance.

Positive and negative enrichments of knockout genes were performed to identify the gene categories that were significantly present or absent in the 3 clusters (Fig. 5). Neither positive nor negative gene categories were enriched in Cluster A, likely due to its small size. Interestingly, Clusters B and C showed completely opposite enrichment patterns, meaning that if a gene category was positively enriched (i.e., significantly abundant) in Cluster B (Fig. 5, left), it was negatively enriched (i.e., notably rare) in Cluster C (Fig. 5, right), and vice versa. This strongly suggests that these gene categories influence bacterial population dynamics in divergent ways. The significant presence of the enzyme (e) in Cluster B indicates that knocking out genes encoding enzymes was conditionally lethal to bacterial growth, as shown statistically (Fig. 3). Its significant absence in Cluster C supports this, as the growth curves in Cluster C showed normal population dynamics, resembling the typical S-shape (Fig. 4F). A considerable number of gene categories were positively enriched in Cluster C, indicating they had minor impacts on bacterial population dynamics. Surprisingly, the transporter (t), which was thought to be essential for bacterial growth, was enriched in Cluster C, along with unknown or predicted function genes.

Additionally, GO enrichment analysis showed that only positively enriched GOs were observed in Cluster A, while only negatively enriched GOs were present in Cluster C. The biological processes related to the cell cycle and division were positively enriched in Cluster A, whereas various biosynthetic processes involving vitamins, nucleosides, and amino acids were negatively enriched in Cluster C (Table). This indicated that the GOs positively and negatively enriched in Clusters A and C had moderate and substantial impacts on bacterial population dynamics, respectively. In a word, all these biological processes must have participated in regulating bacterial population dynamics. Both positively and negatively enriched GOs, related to biosynthesis and transport, respectively, were observed in Cluster B (Table). It suggested that a deficiency in biosynthesis was lethal, while one in transport was nonlethal, which aligns with the enriched gene category (Fig. 5).

**Table 1.** Positively and negatively enriched Gene Ontology (GO) terms in the 3 clusters. A, B, and C indicate the 3 clusters. “All” and “N” indicate the number of genes assigned to the corresponding GO term and the number of genes assigned to the cluster, respectively. “FC” and “FDR” represent the fold change and the statistical significance of the enrichment, respectively. An increase or decrease in enrichment is indicated by “+” or “−”, respectively.

Cluster	GO terms	All	N	FC	+/−	FDR
A	Cell division	13	4	52.04	+	0.002
A	Cell cycle	16	4	42.29	+	0.002
B	“De novo” IMP biosynthetic process	7	7	11.84	+	6E−5
B	Proline biosynthetic process	3	3	11.84	+	0.03
B	Tryptophan biosynthetic process	5	5	11.84	+	0.001
B	“De novo” UMP biosynthetic process	7	7	11.84	+	6E−5
B	Histidine biosynthetic process	8	8	11.84	+	1E−5
B	Pantothenate biosynthetic process	5	5	11.84	+	0.001
B	“De novo” NAD biosynthetic process from aspartate	3	3	11.84	+	0.03
B	Serine biosynthetic process	3	3	11.84	+	0.03
B	Arginine biosynthetic process	8	8	11.84	+	1E−05
B	Leucine biosynthetic process	7	6	10.15	+	0.0006
B	“De novo” pyrimidine nucleobase biosynthetic process	7	6	10.15	+	0.0006
B	Biotin biosynthetic process	6	5	9.87	+	0.003
B	Chorismate biosynthetic process	6	5	9.87	+	0.003
B	Glutamine metabolic process	11	9	9.69	+	1E−5
B	Pyridoxine biosynthetic process	5	4	9.47	+	0.05
B	Methionine biosynthetic process	7	5	8.46	+	0.005
B	Aldehyde biosynthetic process	6	4	7.89	+	0.02
B	Macromolecule metabolic process	252	10	0.47	−	0.03
B	Carbohydrate metabolic process	113	2	0.21	−	0.04
B	Transmembrane transport	197	0	<0.01	−	5E−7
B	Nitrogen compound transport	82	0	<0.01	−	0.01
B	Organic substance transport	139	0	<0.01	−	9E−5
B	Response to chemical	111	0	<0.01	−	0.001
B	Monoatomic ion transport	66	0	<0.01	−	0.04
C	Water-soluble vitamin biosynthetic process	25	9	0.4	−	0.05
C	Aromatic amino acid family biosynthetic process	14	2	0.16	−	0.02
C	Ribonucleoside monophosphate biosynthetic process	19	2	0.12	−	0.0006
C	Pyrimidine nucleoside monophosphate biosynthetic process	8	0	<0.01	−	0.05
C	Histidine biosynthetic process	8	0	<0.01	−	0.04
C	Pyrimidine nucleotide biosynthetic process	8	0	<0.01	−	0.05
C	Arginine biosynthetic process	8	0	<0.01	−	0.04

#### Enriched functions in growth curves with high or low experimental reproducibility

**Fig. 6.**
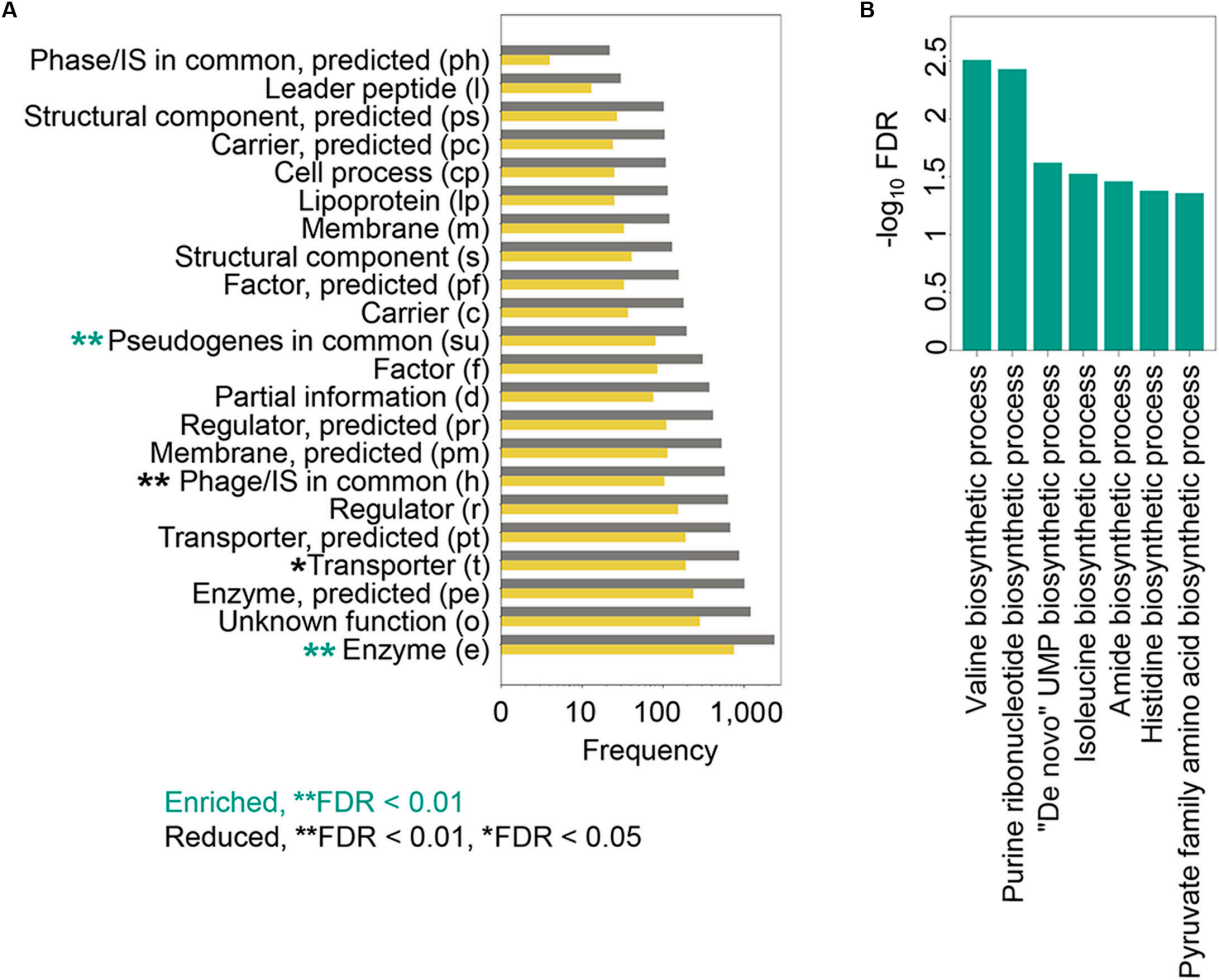
Functional enrichment of growth curves before and after data cleaning. (A) Count of growth curves assigned to 22 gene categories. Gray and yellow bars show the number of growth curves before and after data cleaning, respectively. Asterisks denote the level of statistical significance. Green and black asterisks represent positive and negative enrichment, respectively. (B) Positively enriched GO terms. Statistical significance is indicated on a logarithmic scale.

Thousands of growth curves were removed from the analysis during data cleaning (Fig. 4). The impact of this step on the enrichment analysis was further evaluated. The number of knockout genes assigned to 22 gene categories was tallied, and the distributions of all data versus cleaned data were compared (Fig. 6A). Results indicated that data cleaning significantly enriched the gene categories of e and su (Fig. 6A, green asterisks) and significantly removed those of t and h (Fig. 6A, black asterisks). This suggests that growth curves of *E. coli* strains with knockout genes encoding enzymes and pseudogenes were highly reproducible, whereas those related to transport and insertion sequences had lower reproducibility. Additionally, biological processes associated with biosynthesis were notably enriched after data cleaning (Fig. 6B), while no enrichment was observed in the growth curves of knockout genes that were removed. That is, the growth curves of *E. coli* strains with knockout genes involved in biosynthesis showed considerably lower experimental variation than others.

The variability in growth curves among experimental replicates probably reflected the extent of biological fluctuation caused by the knockout gene rather than the experimental manipulation. To test this hypothesis, the prefiltering variance across all gene categories was additionally evaluated (Fig. S2 and Table S12). Changes in specific functional categories inherently led to higher replicate variation; e.g., transporters (t) exhibited higher baseline shape variation than enzymes (e). As the data cleaning strategy strictly selected for highly reproducible trajectories, it inherently filtered out a higher proportion of low-reproducible shapes of curves. While some minor categories exhibited even higher variance, their small sample sizes precluded statistical significance after correction. Thus, the specifically enriched biological functions in the cleaned data suggested that their conservation involved the knockout genes. It was supported by the enrichment of entire branched-chain amino acid (Leu, Val, and Ile) biosynthetic processes (Fig. 6B), as they were broadly conserved across the bacterial tree of life [50–52] and were demonstrated to be a general regulator for bacterial growth parameters [15]. Conversely, those that were significantly depleted in the cleaned data indicated their dispensable role in living cells. The findings of an increase in enzyme and biosynthesis activities and a decrease in phage and insertion sequences (Fig. 6) were biologically reasonable given their importance in known mechanisms [53,54].

To verify that the data cleaning strategy (retaining the top 25% of similarities) did not introduce any bias in the analysis, a systematic sensitivity analysis was also performed by adjusting the inclusion thresholds to the top 10%, 20%, 30%, and 50% (Table S13). The main patterns of functional enrichment remained very consistent across strict to moderate thresholds. Specifically, from the 10% to 30% thresholds, the significant positive enrichment of enzymes (e) and the significant depletion of predicted transporters (t) were reliably maintained (false discovery rate [FDR] < 0.05). However, when the threshold was expanded to 50%, the depletion signal for transporters no longer reached statistical significance (FDR = 0.225). This suggests that overly lenient thresholds allow the inclusion of high-variance, poorly reproducible growth curves that can mask true biological signals. These results confirmed that the 25% cutoff is robust, as it best balances sample retention with the necessary stringency to detect genuine biological growth patterns.

## Discussion

The importance of this study lies in its innovative methodology and conceptual approach. First, we classified bacterial growth curves using time series machine learning algorithms originally developed for applications such as speech recognition and pattern matching [55,56]. Second, we tested the linkage between growth patterns and biological functions using 2 distinct approaches: one based purely on statistical criteria and the other incorporating replication variance from biological experiments. Both methods revealed significant associations between growth patterns and biological functions, particularly with respect to specific gene categories (such as enzymes) and biosynthetic processes (Figs. 3 and 5 and Table 1). The substantial overlap in the linkages identified by both approaches not only demonstrates the reliability of the results but also provides initial validation that growth curves can capture genetic changes, such as those resulting from single-gene knockouts. While the enrichment of biosynthetic genes (Fig. 6) reflected auxotrophic defects, the DTW/DDTW framework based on the shape of growth curves was not restricted to a simple binary classification of growth versus nongrowth. Unlike traditional parameter-based models that lost high-dimensional information (Fig. S1), this shape-based method identified multiple growing subclusters (e.g., distinct normal-like trajectories in Fig. 4F). It showed that the DTW/DDTW framework effectively captured subtle kinetic variations, such as small shifts in stationary phases or deceleration rates, which rigid parameter fitting could not achieve. Furthermore, since experimental replicates are essential in biology to account for variance arising from biological noise [57,58] and manipulation errors [35,36], we leveraged the variance between replicates as an analytical criterion. The severe cluster imbalance (e.g., the massive C0 cluster) somewhat properly mirrored the biological reality that most single-gene knockouts were phenotypically silent. Compared to centroid-based algorithms (e.g., K-means or Ward) that force artificial fragmentation, the clustering method used here (i.e., Average, unweighted pair group method with arithmetic mean [UPGMA]) preserved the near-WT baseline while isolating genuine defects (Table S14). This approach not only mitigates bias from unavoidable experimental errors but also capitalizes on the information provided by experimental replicates. As high-throughput experimental data become increasingly available, the concepts and methods explored in this study offer a foundational framework for large-scale analyses aimed at uncovering inherent biological functions and dynamic population behaviors.

The present data mining approach successfully identified biologically meaningful associations, particularly regarding the functional essentiality for bacterial population dynamics. Whether using statistical criteria or considering experimental replicates, functional enrichment analyses of knockout genes consistently highlighted biosynthetic processes, especially those related to amino acid biosynthesis (Fig. 3E, Table). This prominent pattern may be attributable to the use of a minimal growth medium lacking nutritional supplements, which accentuates the impact of biosynthetic gene knockouts on growth dynamics. The substantial influence of biosynthetic processes on bacterial growth has also been observed in bioproduction studies [59–61]. Notably, enrichment was not uniform across all 20 amino acids; the biosynthetic pathways for Pro, Met, Leu, Trp, His, and Arg were highly significantly enriched regardless of the analytical method (Fig. 3E and Table), suggesting that these amino acids play a particularly important role in shaping bacterial population dynamics. While phenotypic analyses of the Keio collection have been extensively explored in previous seminal studies using predefined quantitative parameters [62–64], the parameter-free global shape alignment (Fig. 2 and Fig. S1) and replicate variance consideration (Fig. 4 and Fig. S2) provided alternative dimensions of biological insight. For instance, while the effects of Leu, Trp, and Pro on individual growth parameters were previously known [15,24], the prominent roles of Met, Arg, and His biosynthetic processes in influencing overall growth dynamics were newly observed. These findings provide valuable insights for systems metabolic engineering and the development of whole-cell simulations. Note that the functional associations identified here were highly influenced by environmental conditions. Testing representative priority knockouts in a different growth medium (Fig. S3) showed that growth curves and their clusters could change substantially with shifts in the nutritional landscape, confirming the medium-specific nature of clustering. However, because the DTW/DDTW analytical framework focuses purely on phenotypic shape without considering genetic background, it remains applicable to other microbial strains and environmental settings for future research.

This study has several limitations related to both the machine learning methods and the experimental dataset. The precision of growth curve classification remains an area for improvement. When clustering growth curves using only statistical criteria, the SC decreased rapidly as the number of clusters increased (Fig. 2B), indicating that this approach struggled to distinguish subtle differences between growth curves and thus provided only a coarse classification. This limitation may stem from the specific machine learning techniques used, particularly the combination of DTW and DDTW, which may require further fine-tuning to effectively capture fine-grained differences. Future work should consider employing more advanced algorithms or enhanced feature extraction methods to improve classification precision [65]. Additionally, the growth curve dataset, based on OD_600_ measurements from biological experiments, inherently contains experimental errors, which are common in biological research [35,36,66]. Although experimental procedures were carefully optimized for accuracy [67], the dataset’s precision may still be insufficient to detect subtle effects of gene knockouts. Furthermore, since OD_600_ measurements are affected by factors like cell size and morphology [5,68], the observed growth dynamics may not fully represent actual population changes, as noted previously [69]. However, by using DDTW, the analytical framework naturally reduces such bias by emphasizing the rate of change at each moment instead of relying on the absolute OD_600_ values. Further improvements in experimental assay methods and increased numbers of experimental replicates may be necessary to achieve a higher-resolution connection between biological functions and growth curves.

In summary, this study investigated whether patterns in bacterial growth curves can be linked to underlying biological functions, demonstrating the potential of time series data mining to uncover complex genetic influences on bacterial population behavior. We established connections between growth curves and biological functions at the levels of gene categories and metabolic pathways (Figs. 3 and 5 and Table). While these connections are still approximate, our findings suggest that it is possible to correlate aspects of bacterial population dynamics with genetic information. It is important to note that the current analytical framework relies on unsupervised pattern discovery and functional enrichment to uncover these associative links, rather than employing a supervised predictive model. Further exploration of the roles of individual genes, gene categories, or pathways at different stages of the growth curve could lead to a more comprehensive understanding of how these elements govern bacterial population behavior. Future studies incorporating supervised classification tasks and rigorous cross-validation metrics will be essential to transition from descriptive association to true predictive modeling. As larger biological datasets become available, data mining strategies will play an increasingly important role in elucidating the dynamic patterns of living systems in both life sciences and systems biotechnology.

## Materials and Methods

### Strains and growth medium

The Keio collection, comprising 3,909 single-gene knockout strains [30], was distributed from the National BioResource Project, National Institute of Genetics (Shizuoka, Japan). These strains were all cultured in the minimal medium M63, as previously described [28].

#### Growth curves

The *E. coli* strains were cultivated in M63 minimal medium following the method outlined in our previous studies [28,67]. The growth of the strains was monitored by measuring the absorbance at 600 nm (OD_600_), with readings taken every 15 min over a period ranging from 24 to 48 h. This process was repeated 3 times independently for each strain, ensuring consistency and reliability of the results. The dataset comprising 11,727 growth curves, 3 experimental replicates of 3,909 strains, was adopted in this study. The original datasets and experimental details are described elsewhere [28,37].

#### Data preprocessing

The raw growth curve data were preprocessed before time series data analysis. Redundant and unreliable records were removed from the temporal data to ensure that the growth curves ended at similar growth phases. The maximum OD_600_ mean was identified by calculating the average of 5 consecutive OD_600_ values with the highest mean, as previously reported [25,70]. The data points after the 5 points following the maximum OD_600_ mean were truncated to maintain consistency in the stationary phase, ensuring that any complex prestationary dynamics (such as multiphasic growth patterns) were fully preserved for analysis. Growth curves that showed no continuous increase in OD_600_ or failed to reach the maximum OD_600_ were removed from the dataset. Finally, a total of 10,247 growth curves from 3,880 single-gene knockout strains were included in the subsequent analyses (Table S7).

#### DTW and DDTW evaluation

All implementations of DTW [39,40] refer to the fastdtw version 0.3.4 in Python, using Euclidean distance as the standard for calculating distances. For DDTW [41,42], the following equation [Eq. (1)] is used as an approximation to replace the derivative.

$$d_{i}=\frac{\left(x_{i}-x_{i-1}\right)+\frac{\left(x_{i+1}-x_{i-1}\right)}{2}}{2}$$ (1)

The distance calculation employs Euclidean distance, and the approach involves the use of complete dynamic programming. The underlying dynamic programming algorithm employed a Euclidean distance metric. Note that in DDTW, this metric was applied to the transformed sequence of slope averages (*d_i_*) rather than the raw OD_600_ values. The symbol *x_i_* denotes the OD_600_ read of a given growth curve at the time point *i* (*i* = 1, …, *n*). *d_i_* denotes the approximate first derivative (instantaneous rate of change) of the OD_600_ read at *i*. The term (*x_i_* − *x*_*i−*1_) is the backward difference between the current and previous measurements, while (*x*_*i*+1_ − *x*_*i*−1_)/2 is the central difference spanning the adjacent forward and backward points. The unit of *d_i_* is OD_600_ per unit time. The resulting derivative sequence *d_i_* is used as the input for DDTW to capture shape/gradient similarity between growth curves. The combination of DTW and DDTW for creating a similarity matrix to cluster the curves is as follows [Eq. (2)].

$$d\;\left(X,Y\right)=\left(1-\alpha \right)\;\mathrm{DTW}\left(X,Y\right)+\alpha \;\text{DDTW}\left(X,Y\right),\alpha \in \left[0,1\right]$$ (2)

*X* and *Y* denote 2 observed growth curves sampled at time points *i* (*i* = 1, …, *n*). DTW(*X*, *Y*) and DDTW(*X*, *Y*) are the distance measures computed by DTW on the raw OD_600_ traces and by DDTW on the derived derivative sequences *d_i_* [as defined in Eq. (1)], respectively. The scalar *α* ∈ [0, 1] is a tunable hyperparameter that controls the relative contribution of the 2 distance terms. Before applying this linear combination, it is essential to account for the inherent difference in scale between DTW and DDTW distances. In our dataset, the global mean of the DDTW distance matrix was 1/1,688 of the DTW matrix. Therefore, a global linear scaling factor was applied, multiplying all DDTW distances by 1,688 to strictly align their average magnitudes. This normalization ensures that the hyperparameter *α* functions as a mathematically sound proportional weight rather than being dominated by the larger absolute values of DTW.

#### Data cleaning

Pairwise distances between any pair of growth curves within the experimental replicates were calculated using both DTW and DDTW, as described above. This resulted in 2 or 3 pairwise distances for both DTW and DDTW per replicate set. Across the dataset, the thresholds for high similarity were set based on the top 25% of the most similar pairs (those with the lowest distances), specifically DTW < 1.105 and DDTW < 0.000506. Any growth curve involved in a pair that met its respective threshold was retained. Ultimately, only the growth curves that satisfied both the DTW and DDTW top-25% criteria simultaneously were used for downstream analysis.

#### Clustering analysis

Hierarchical clustering was conducted using the Average linkage (UPGMA) method via the fcluster and linkage functions from SciPy version 1.10.0 in Python. The number of clusters was determined by the elbow rule using a multimetric approach, including the SC and the Davies–Bouldin Index [71]. According to the elbow method, as the number of clusters increases, the error typically decreases rapidly in the initial stages, after which the rate of decrease slows down. The point where the rate of decrease slows is considered the optimal number of clusters. As the number of clusters increased, the SC initially declined slowly and then dropped sharply. This turning point resembled identifying the “knee point” (the point of maximum curvature before a steep decline), which we referred to as a “reverse elbow” in this study. The point just before this sharp drop was considered the optimal number of clusters. The raw data of SC values are summarized in Table S4.

#### Enrichment analysis

A total of 22 gene categories [72], which contain more than 10 genes, were used for the analysis, as previously reported [73,74]. To optimize statistical power, sparsely populated categories lacking significant enrichment were excluded from the FDR correction. The enrichment was performed according to the hypergeometric probability, which is calculated using the following formula [Eq. (3)].

PX=k=Kk·N−Kn−kNn (3)

*N* denotes the total number of knockout genes in the current dataset, *K* indicates the number of knockout genes in the category of interest within that dataset, *n* represents the number of knockout genes assigned to the cluster (sample size), and *k* denotes the number of genes from the category of interest observed in that cluster. GO enrichment was according to the GO website (http://www.geneontology.org) [75,76]. The “Analyzed List” consisted of the knockout genes in each cluster, and the “Reference List” comprised all knockout genes across all clusters. Fisher’s exact test was used to determine the significance of enrichment, and FDR correction was applied to the *P* values using the Benjamini–Hochberg procedure to account for multiple testing (FDR < 0.05). No minimum gene count filter was applied prior to the enrichment test. To ensure a comprehensive functional landscape, all 22 established categories were visualized. Although testing all 22 categories introduces a multiple-testing penalty, the hypothesis space is sufficiently small that the Benjamini–Hochberg FDR correction does not severely compromise statistical power or mask significant biological signals.

## Data Availability

All data generated or analyzed in this study are available within the paper and its Supplementary Tables. The code is available on GitHub at the following link: https://github.com/loutakuki/data-mining-bacterial-growth-dynamics.
