# Supplementary material for: Time Series Data Mining for Linking the Shape of Bacterial Growth Curves to Biological Functions
Source: Comput Struct Biotechnol J. 2026 Apr 1;35(1):0029. doi: 10.34133/csbj.0029 (PMC13067955; doi:10.34133/csbj.0029)
Supplement: Supplementary 1 — Figs. S1 to S3 Tables S1 to S14 [file csbj.0029.f1.pdf]

Supplementary information

Time series data mining for linking the shape of bacterial growth curves to biological functions

Zehui Lao[1] and Bei-Wen Ying[1,2,*]

[1]School of Life and Environmental Sciences, University of Tsukuba, 1-1-1 Tennodai, Tsukuba, 305-8572 Ibaraki, Japan
[2]MiCS, University of Tsukuba, 1-1-1 Tennodai, Tsukuba, 305-8572 Ibaraki, Japan

*Corresponding: ying.beiwen.gf@u.tsukuba.ac.jp

Supplementary figures (Figures S1~S3) **p. 2~4**

Supplementary tables (Tables S12~S14, small tables) **p. 5~7**

Supplementary table captions (Tables S1~S11, large dataset) **p. 8~9**

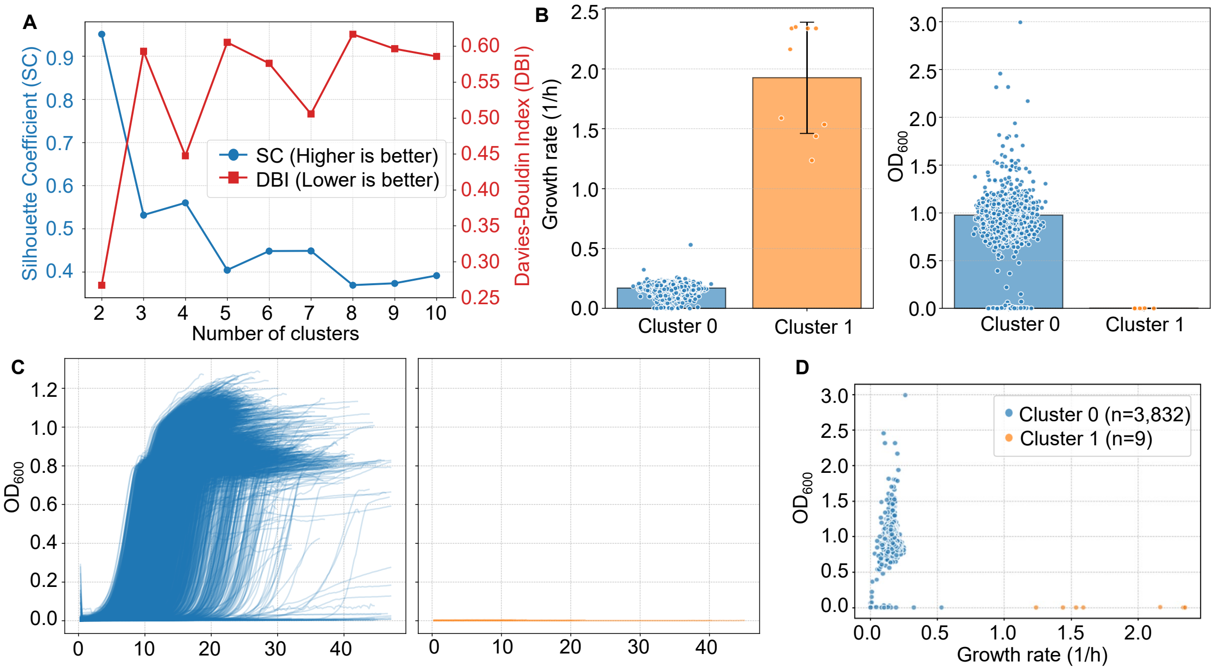

Figure S1. Parameter-based clustering of bacterial growth curves. A. Line chart illustrating the clustering evaluation metrics across different numbers of clusters (k = 2 to 10). K-Means clustering was performed using the standardized maximum specific growth rate and OD_{600} extracted via the Gompertz model. The blue line (left Y-axis) represents the Silhouette Coefficient (SC, where higher is better), and the red line (right Y-axis) represents the Davies-Bouldin Index (DBI, where lower is better). The optimal mathematical separation occurs at k = 2, indicating a severe lack of resolution for subtle kinetic substructures. **B.** Bar charts showing the distribution of the extracted growth rate (left) and maximal OD_{600} (right) for the two clusters identified at k = 2. Bars represent the mean values, and error bars indicate standard deviations. Individual data points for all 3,880 knockout strains are overlaid as jittered scatter points to visualize the raw data distribution. **C.** Original growth trajectories grouped by the parameter-based K-Means clustering (k = 2). Transparent lines represent individual growth curves assigned to Cluster 0 (left) and Cluster 1 (right). **D.** Scatter plot showing the relationship between growth rate and maximal OD_{600} for the 3,880 single-gene knockout strains. Dots are colored based on their cluster assignment from the Gompertz model (k = 2).

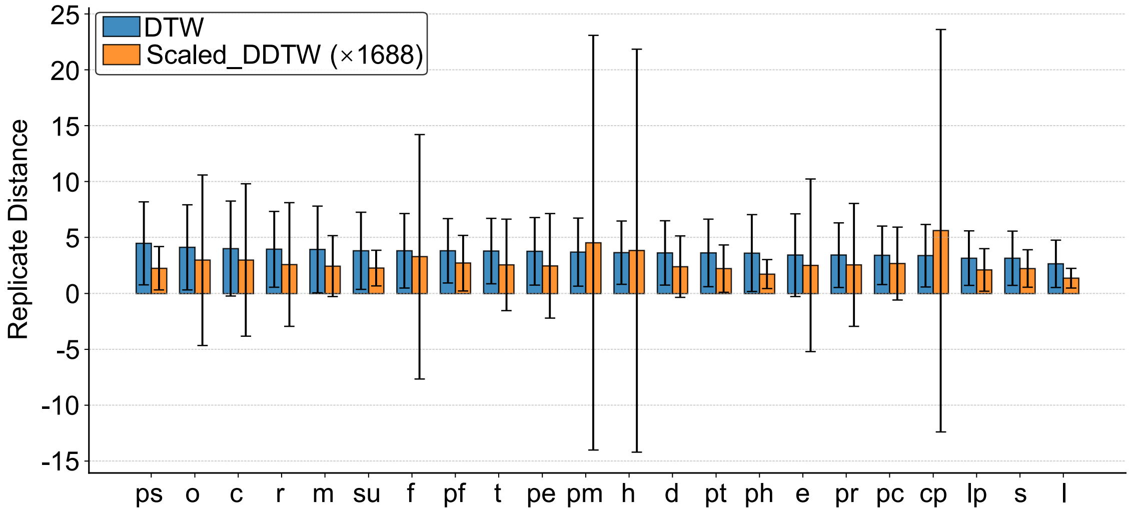

Figure S2. Inherent replicate variability across functional gene categories prior to data cleaning. The bar chart illustrates the baseline phenotypic instability, quantified as the average distance between experimental replicates, for single-gene knockout strains grouped by their respective functional categories. The blue bars represent the mean Dynamic Time Warping (DTW) distances, and the orange bars represent the mean Derivative Dynamic Time Warping (DDTW) distances. To enable direct visual comparison on the same axis, the raw DDTW values were scaled by a factor of 1,688. Error bars indicate the standard deviations within each category. Categories on the X-axis are sorted in descending order of their mean DTW distance. Higher distance values signify greater inherent biological noise or morphological fluctuation across experimental replicates. This quantitative variance inherently explains why strict reproducibility-based data cleaning selectively retains specific functional classes over others.

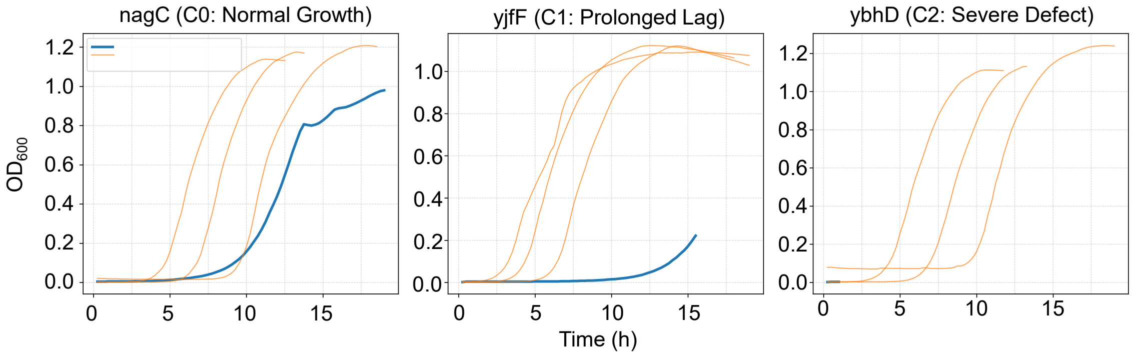

Figure S3. Medium-dependent phenotypic plasticity of representative strains. To demonstrate that the observed kinetic trajectories and subsequent cluster assignments are strictly condition-specific, three representative knockout strains were selected from the primary statistically identified clusters in M63 minimal medium: nagC (representing Cluster C0: Normal Growth), yjfF (representing Cluster C1: Prolonged Lag), and ybhD (representing Cluster C2: Severe Defect/Lethality). The thick blue lines represent their growth trajectories in the nutrient-poor M63 minimal medium (the baseline condition analyzed in this study). The thin orange lines represent their growth trajectories when cultivated in the nutrient-rich Luria-Bertani (LB) medium, with three independent experimental replicates shown for each strain. Notably, the severe growth defect (*ybhD*) and the extensively prolonged lag phase (*yjfF*) observed in the minimal medium were completely rescued in the rich medium, where they exhibited normal, near-wild-type growth dynamics similar to *nagC*.

Table S12. Statistical summary of pre-filtering replicate distances across functional gene categories. Quantitative metrics for within-replicate consistency were applied before data filtering. The number of evaluated genes (Gene_count) and that of replicate pairs (Replicate_Pairs) are indicated for each category, alongside the mean (_Mean) and standard deviation (_Std) for raw DTW and DDTW distances and the scaled DDTW (Scaled_) by multiplying a factor of 1,688.

Category	Gene_ Count	Replicate _Pairs	DTW_ Mean	DTW _Std	DDTW _Mean	DDTW _Std	Scaled_ DDTW _Mean	Scaled_ DDTW _Std
ps	34	104	4.49	3.71	1.E-03	1.E-03	2.26	1.94
o	425	1,063	4.13	3.81	2.E-03	5.E-03	2.98	7.63
c	65	149	4.02	4.24	2.E-03	4.E-03	2.99	6.81
r	213	555	3.95	3.40	2.E-03	3.E-03	2.59	5.53
m	41	115	3.94	3.87	1.E-03	2.E-03	2.45	2.73
su	46	366	3.82	3.45	1.E-03	9.E-04	2.27	1.60
f	106	274	3.82	3.34	2.E-03	6.E-03	3.29	10.93
pf	58	138	3.82	2.87	2.E-03	1.E-03	2.71	2.49
t	298	789	3.79	2.93	2.E-03	2.E-03	2.57	4.09
pe	349	895	3.77	3.02	1.E-03	3.E-03	2.47	4.67
pm	185	457	3.70	3.05	3.E-03	1.E-02	4.54	18.55
h	197	485	3.66	2.83	2.E-03	1.E-02	3.84	18.02
d	129	331	3.63	2.87	1.E-03	2.E-03	2.40	2.75
pt	231	615	3.62	3.02	1.E-03	1.E-03	2.22	2.12
ph	6	12	3.62	3.43	1.E-03	8.E-04	1.73	1.29
e	817	2,005	3.44	3.70	1.E-03	5.E-03	2.52	7.72
pr	143	361	3.43	2.90	2.E-03	3.E-03	2.57	5.50
pc	38	88	3.41	2.62	2.E-03	2.E-03	2.67	3.25
cp	36	102	3.38	2.79	3.E-03	1.E-02	5.63	17.99
lp	40	100	3.16	2.44	1.E-03	1.E-03	2.11	1.91
s	43	121	3.16	2.42	1.E-03	1.E-03	2.24	1.67
l	9	21	2.65	2.11	8.E-04	5.E-04	1.36	0.88

Table S13. Sensitivity analysis of data cleaning thresholds on functional enrichment outcomes. Data retention thresholds represent the top 10%, 20%, 25%, 30%, and 50% of DTW and DDTW similarities. For each threshold, the total number of retained independent growth curves is provided. The analysis specifically tracks the two major functional categories identified in this study: enzymes (e) and predicted transporters (t). For each category, the table details: the ratio of retained curves to the total background curves (k/K), the overall enrichment trend (Enriched [+] or Depleted [-]), and the statistical significance represented by False Discovery Rate (FDR) corrected p-values calculated via the hypergeometric probability test.

Threshold	Retained _Curves	e_Count (k/K)	e_Trend	e_FDR	t_Count (k/K)	t_Trend	t_FDR
10%	636	318/2379	Enriched (+)	7.96E-52	29/872	Depleted (-)	0.000254
20%	1842	590/2379	Enriched (+)	1.12E-21	125/872	Depleted (-)	0.00402
25%	2575	735/2379	Enriched (+)	3.13E-13	189/872	Depleted (-)	0.009027
30%	3325	878/2379	Enriched (+)	9.99E-08	247/872	Depleted (-)	0.005716
50%	6134	1484/2379	Enriched (+)	2.20E-03	511/872	Depleted (-)	0.225154

Table S14. Comparison of different clustering algorithms and linkage methods. The clustering performance (measured by the Silhouette Coefficient) and cluster distribution (shown by the Maximum Cluster Size) across four distinct clustering algorithms, i.e., Agglomerative Clustering with Average (UPGMA), Ward, and Complete linkages, as well as K-Means, are summarized. The evaluation was conducted on the combined DTW/DDTW distance matrix using the optimal parameters ($\alpha = 0.3$, $k = 7$) used in this study.

Clustering Algorithm	Silhouette Coefficient	Maximum Cluster Size
Agglomerative (Average)	0.7967	3,758
Agglomerative (Ward)	0.3685	1,589
Agglomerative (Complete)	0.5141	3,623
K-Means	0.3896	1,228

Supplementary table captions

Table S1. 3,880 processed growth curves. It shows temporal changes in OD_{600} readings of 3,880 single-gene knockout strains grown in M63. Readings are recorded every 15 minutes, and data processing is detailed in Materials and Methods. Gene ID and gene name are provided.

Table S2. DTW matrix of 3,880 growth curves. Pairwise similarities among 3,880 growth curves were evaluated by DTW distances, shown in the matrix format.

Table S3. DDTW matrix of 3,880 growth curves. Pairwise similarities among 3,880 growth curves were evaluated by DDTW distances, shown in the matrix format.

Table S4. SC matrix. The SC (silhouette coefficient) values obtained under different combinations of α and n, which represent the weighting ratio between DTW and DDTW (Eq. 2) and the number of clusters, respectively.

Table S5. Changes in SC regarding the increase in the number of clusters. The changes in SC were calculated by subtracting the SC obtained with n + 1 clusters from that with n clusters.

Table S6. Growth curve clusters based on statistical criteria. 3,880 growth curves were grouped according to changes in SC at the combination of $n = 7$ and $\alpha = 0.3$. The seven clusters are numbered from 0 to 6, and the growth curves are labeled with Gene ID and gene name.

Table S7. 10,247 growth curves. A total of 10,247 processed growth curves from 3,880 single-gene knockout strains are shown. Gene ID and gene name are provided. The suffix after the gene name indicates the three experimental replicates: no suffix, 1, and 2. Data are recorded at 15-minute intervals, and data processing methods are described in Materials and Methods.

Table S8. DTW distances of growth curves between experimental replicates. Pairwise similarities of biological replicates were calculated using DTW. The suffixes indicate which two replicates are being compared (e.g., aaeA_1_2 represents the similarity between the aaeA knockout strain in the first and second replicates)**.**

Table S9. DDTW distances of growth curves between experimental replicates. Pairwise similarities of biological replicates were calculated using DDTW. The suffixes indicate which two replicates are being compared (e.g., aaeA_1_2 represents the similarity between the aaeA knockout strain in the first and second replicates).

Table S10. 2,635 growth curves. A subset of 2,635 growth curves with the top 25% similarity between replicates, determined by both DTW and DDTW, is summarized. The growth curves are labeled with Gene ID and gene name. The suffix after the gene name indicates the three experimental replicates: no suffix, 1, and 2.

Table S11. Growth curve clusters based on experimental replicates. 2,635 growth curves were grouped by similarity across replicates at $n = 3$ and $\alpha = 0.5$. The three clusters are labeled A, B, and C, with each growth curve tagged by Gene ID and gene name. The suffix after the gene name indicates the replicate, with no suffix, 1, or 2.
